# Effect of Immune Cell Infiltration on Occurrence of Pulmonary Hypertension in Pulmonary Fibrosis Patients Based on Gene Expression Profiles

**DOI:** 10.3389/fmed.2021.671617

**Published:** 2021-07-07

**Authors:** Feng Zhu, Lili Zuo, Rui Hu, Jin Wang, Zhihua Yang, Xin Qi, Limin Feng

**Affiliations:** ^1^Graduate School, Tianjin University of Traditional Chinese Medicine, Tianjin, China; ^2^Department of Traditional Chinese Medicine, Hebei North University, Zhangjiakou, China; ^3^Department of Cardiology, Tianjin Union Medical Center, Tianjin, China; ^4^Department of Neonatal, ZiBo Maternal and Child Health Hospital, Zibo, China; ^5^Center for Drug Monitoring and Evaluation Department, Center for Drug Monitoring and Evaluation in Zhangjiakou, Zhangjiakou, China; ^6^Department of Cardiovascular Disease, ZiBo Hospital of Traditional Chinese Medicine, Zibo, China; ^7^First Teaching Hospital of Tianjin University of Traditional Chinese Medicine, Tianjin, China; ^8^Department of Cardiology, The Second Affiliated Hospital of Tianjin University of Traditional Chinese Medicine, Tianjin, China

**Keywords:** pulmonary fibrosis, immune cell infiltration, pulmonary hypertension, random forest classifier, prediction

## Abstract

Pulmonary hypertension (PH) is a frequent complication in patients with pulmonary fibrosis (PF), whereas the mechanism was not well-understood. This study aimed to explore the influence of immune cell infiltration on PH status based on the genomic expression profiles. Microarray data of GSE24988 were downloaded from the GEO database, including 116 lung tissue samples derived from PF patients with various PH status. Proportion of infiltrated immune cells was evaluated using CIBERSORT, a gene expression-based de-convolution algorithm. A random forest classifier was constructed and out of bag (OOB) cross-validation was carried out for PH prediction. The proportions of immune infiltration cells varied differently in PH samples except T regulatory cells (*p*-value = 0). Compared with non-PH samples, increased number of naive B cells and plasma cells were identified in PH samples, whereas activated dendritic cells and M2 macrophages were relatively lower (*p* < 0.05). In the random forest model, these four types of immune cells obtained a higher variable importance score than other cells, including mean decreased accuracy and mean decreased gini evaluation. We ran the OOB cross-validation in each sample of datasets (training set and testing set) and obtained 79 and 69% accuracy, respectively. Abnormal proportions of four types of immune cells were identified in PH samples compared with non-PH samples, suggesting their involvement in PH development. In summary, the immune cell infiltration in PF patients is associated with the PH status of patients, which deserves further investigation in the future.

## Introduction

Pulmonary hypertension (PH) is a frequent complication in patients with chronic lung diseases such as idiopathic pulmonary fibrosis (IPF) and chronic obstructive pulmonary disease (COPD). Previous studies revealed that the prevalence of PH in IPF patients ranged from 14 to 84% in the United States (1, 2). The presence of PH is strongly correlated with high mortality of chronic lung diseases. The pathogenesis of PH is initiated by formation of scars in lung tissues and severe vascular remodeling, causing pressure to smallest arteries and increasing resistance to blood flow (3). Unlike systemic hypertension, this condition only affects the arteries of lungs. Subsets of patients will develop to end stage of PF and often refer for lung transplant. PH is defined by a mean pulmonary arterial pressure (mPAP) of ≥20 mm Hg (4) and severe is defined as 35 mm Hg or more (5). Although many studies have been conducted for understanding this disease, patients suffered from PH-associated lung disease carrying poor prognosis. Therefore, more effort aiming at PH treatment should be carried out to decrease the mortality due to chronic lung diseases.

Increased evidences have demonstrated that inflammatory component and immune system function were key factors in the development of PH. For example, the inflammatory cell infiltration in perivascular tissue of PH patients was associated with intima and media remodeling of plexiform lesions (6). These inflammatory cells can release cytokines to directly regulate the microenvironment of vessels and further worsen disease progression. Dendritic cell (DC) recruitment was also identified in muscular pulmonary arteries of PH patients and an experimental PH rat model, which was induced by monocrotaline exposure (7). Denise et al. showed abnormal DC subset distribution and activation status were involved in pathogenesis of pulmonary arterial hypertension (8). Furthermore, increased IL-6 level in serum produced by DCs and pulmonary vascular immune cell infiltration suggested a major role of immune system in PH development (9). In addition to immune cells, elevated levels of inflammatory cytokines, such as IL-1, IL-6, etc., were also reported in patients with severe PH status (10, 11). These cytokines derived from immune cells were linked with poor survival of patients. Meanwhile, overproduction of several cytokines (CCL2, CX3CL1/CX3CR1, and CCL5) contributed to inflammatory process and pulmonary vessel remodeling in severe PH patients (12–14). Although most studies have explored the prevalence of immune cell infiltration in PH patients, the distribution and proportion were still not well-understood in this disease.

Since the PF patients with severe PH exhibited a poor outcome, it is necessary to investigate whether infiltrated immune cell features represent a reliable phenotype to distinguish this disease. In this study, we analyzed the status of immune cell infiltration in lung tissues based on gene expression profiles, aiming to distinguish PH specimen from non-PH specimen. We observed abnormal proportion of four types of immune cells in PH lung tissue samples compared with non-PH samples, suggesting their involvement in PH development. Increased number of naive B cells and plasma cell infiltration were identified in PH tissue, whereas activated DCs and M2 macrophages were relatively lower. OOB cross-validation in the random forest model was carried out for prediction of PH status in each sample, and finally, we obtained a relatively higher accuracy. Our results suggested that this model might be an effective approach for PH prediction.

## Materials and Methods

### Data Resource

The genomic profiles under accession number GSE24988 (15) were downloaded from the NCBI GEO (http://www.ncbi.nlm.nih.gov/geo/) database, including 116 lung tissue samples. In the original dataset, the fresh frozen lung tissue specimens were obtained from the recipient organs of PF patients undergoing lung transplantation, which were snap frozen in liquid nitrogen and stored at −80°C. These specimens were derived from PF patients with various PH status range: non-PH (mPAP ≤ 20 mm Hg), *n* = 22; intermediate PH group (21 mm Hg ≤ mPAP ≤ 39 mm Hg), *n* = 45; severe group (mPAP ≥ 40 mm Hg), *n* = 17. A cohort of PH patients (with consecutive PF) (*n* = 32) were considered as validation set. The dataset was tested on the platform of [HuGene-1_0-st] Affymetrix Human Gene 1.0 ST Array [transcript (gene) version]. For detailed patient information, see Supplementary Table 1.

### Analysis of Immune Cells Infiltration

Based on the gene expression matrix, CICERSORT software can be used to characterize the composition of immune infiltrating cells, according to the 547 preset barcode genes in deconvolution algorithm. We used the CIBERSORT software (16) to calculate the relative proportion and corresponding *p*-value of 22 immune cells in each cancer sample. The sum of all estimated proportions of immune cell types in each sample was equal to 1.

### Statistical Analysis

Subsequently, based on the relative content proportion of 22 immune cells, the correlation between the immune cells was calculated to obtain Pearson correlation coefficient, using R language (version 3.5.2, the same below).

The principal component analysis was conducted on all samples (“princomp” function in R language). The top 2 principal components contributed mostly to the variance were selected to generate scatter plots of the samples.

Based on two-sided *t*-test (*p*-value < 0.1) and the absolute value of the difference <0.01, the differential immune cells between (severe PH patients + moderate PH patients) and (no PH specimens) were identified.

### Construction of Random Forest Classifier for PH Status Prediction

Based on the “randomForest” function package in R language, we selected the parameters ntree = 300, mtry = 5 to construct a random forest model. A random forest classifier (17, 18) was used to predict the PH status in each sample based on immune infiltration class. The immune cell features with mean decreased accuracy more than zero were selected as important factors to construct a random forest model. Patients with intermediate and severe PH were considered as positive samples, while patients with non-PH were taken as negative samples. Then, the model was verified in validation set (*n* = 32).

## Results

### Evaluation of Infiltrating Immune Cells Associated With PH

**Figure 1:**
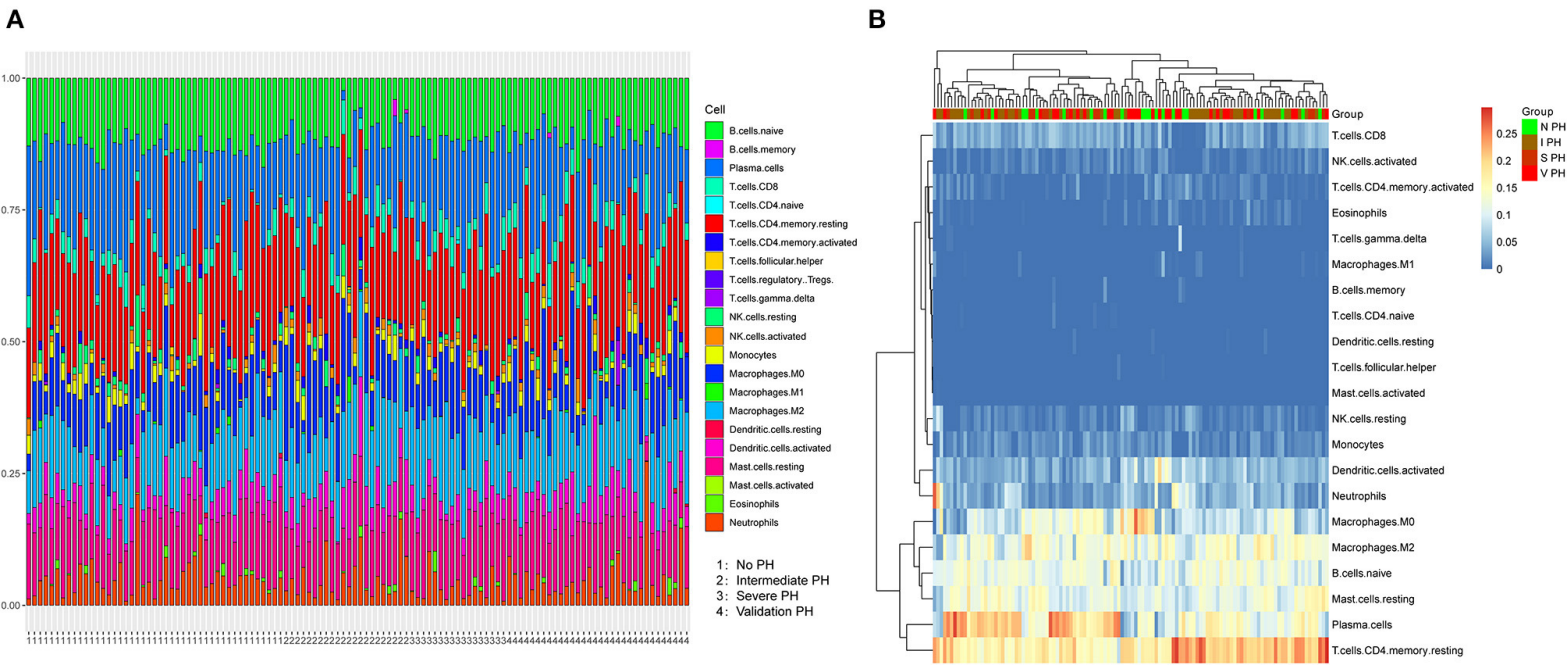
Characterization of immune cell infiltration subsets in PF patients associated with PH. **(A)** The proportions of 21 immune cells varied in samples with PH or non-PH. Horizontal axis represented the patients with non-PH, intermediate PH, severe PH and validated PH. **(B)** Hierarchic clustering analysis was conducted based on 21 infiltrated immune cells in the severe PH (*n* = 17), intermediate PH (*n* = 45), non-PH (*n* = 22), and validated PH (*n* = 7) groups of patients. PF, pulmonary fibrosis; PH, pulmonary hypertension.

After data normalization, the proportions of 22 types of infiltrating immune cells in PF specimens were evaluated using CIBERSORT software (Figure 1). The proportions of immune cells were different in PF samples except regulatory T cells (Figure 1A). The content of regulatory T cells was 0 in all samples; thus, regulatory T cell type was excluded in subsequent analysis. Furthermore, the *p*-values derived from CIBERSORT were <0.05, which indicated that these samples contained a higher immune cell infiltration. Hierarchical cluster analysis was performed on 116 samples based on these infiltrating immune cells, which suggested that individuals with PH status could not be divided from patients without PH diseases based on infiltrating immune cells status (Figure 1B).

### The Difference of Immune Cell Infiltration Between PH and PF Patients

**Figure 2:**
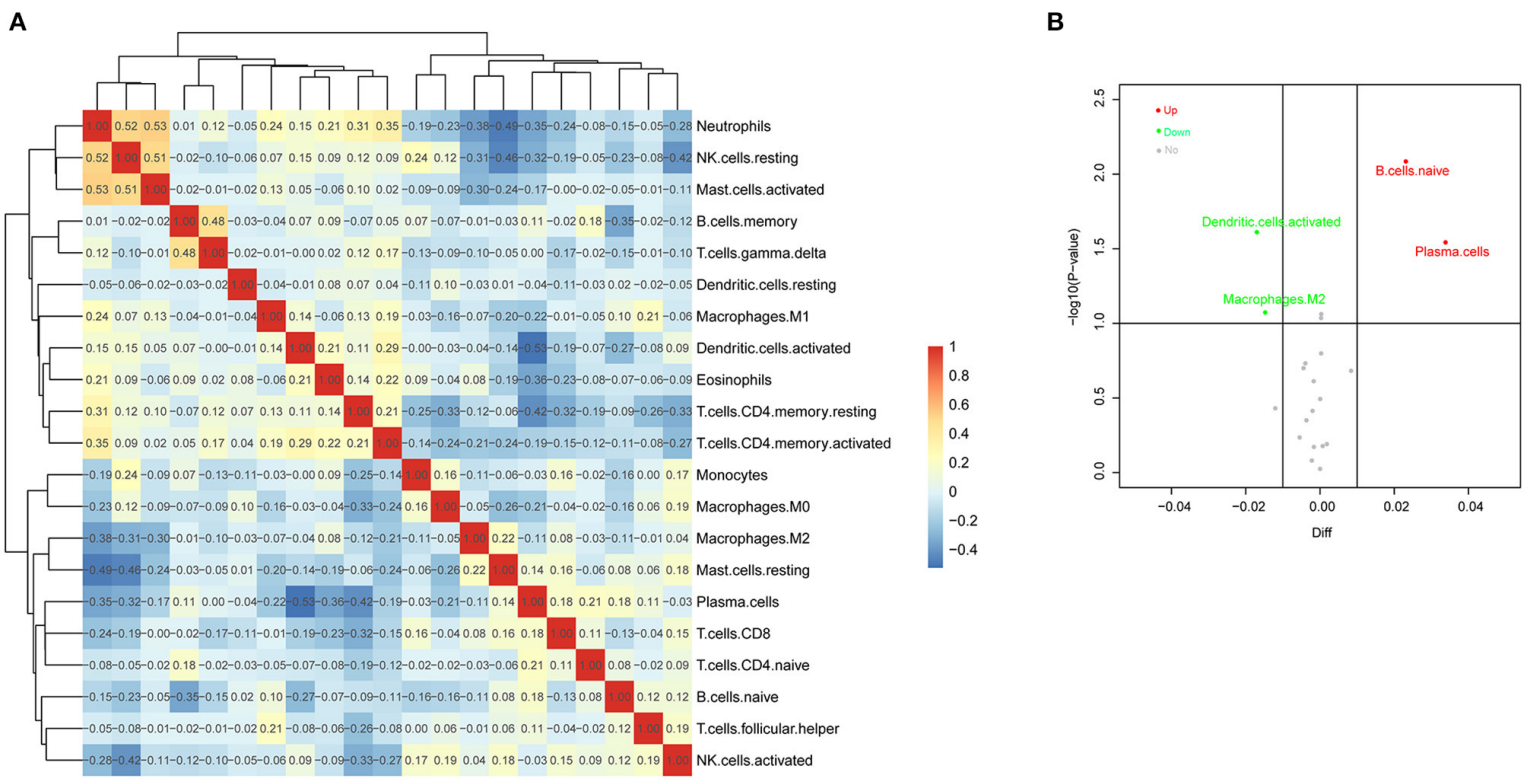
Evaluation of immune cell infiltration in PF patients. **(A)** Correlation matrix of infiltrated immune cell proportion in 116 PF samples. The deeper the red and the blue, the stronger the correlation among them. **(B)** Volcano Plot was used to visualize features of infiltrated immune cells. The gray points referred to the immune cells subsets without significant differences, and the red and blue points refered to the immune cells subsets with significant difference. In addition, red points represented upregulation and green dots represented downregulation. PF, pulmonary fibrosis.

We analyzed the correlation of all 21 immune cell subset proportions among the 116 PF samples and the correlation among 21 immune cells. As shown in Figure 2A, there was no significant difference between the two groups (*p* > 0.5, regardless of positive or negative correlation), and the correlation between 21 immune cells was weak. The results indicated that there was a greater heterogeneity in the infiltration of different immune cells in different patients, which might be closely related to PH progression. Meanwhile, the difference of immune cell infiltration was also evaluated between PH samples and non-PH samples. Interestingly, compared with non-PH samples, PH tissues generally exhibited a higher proportion for naive B cells and plasma cell infiltration, whereas activated DCs and M2 macrophages were relatively lower (Figure 2B). Our results suggested that the proportion of these four types of infiltrated immune cells might be potentially associated with the PH patients.

### Construction of Random Forest Classifier

**Figure 3:**
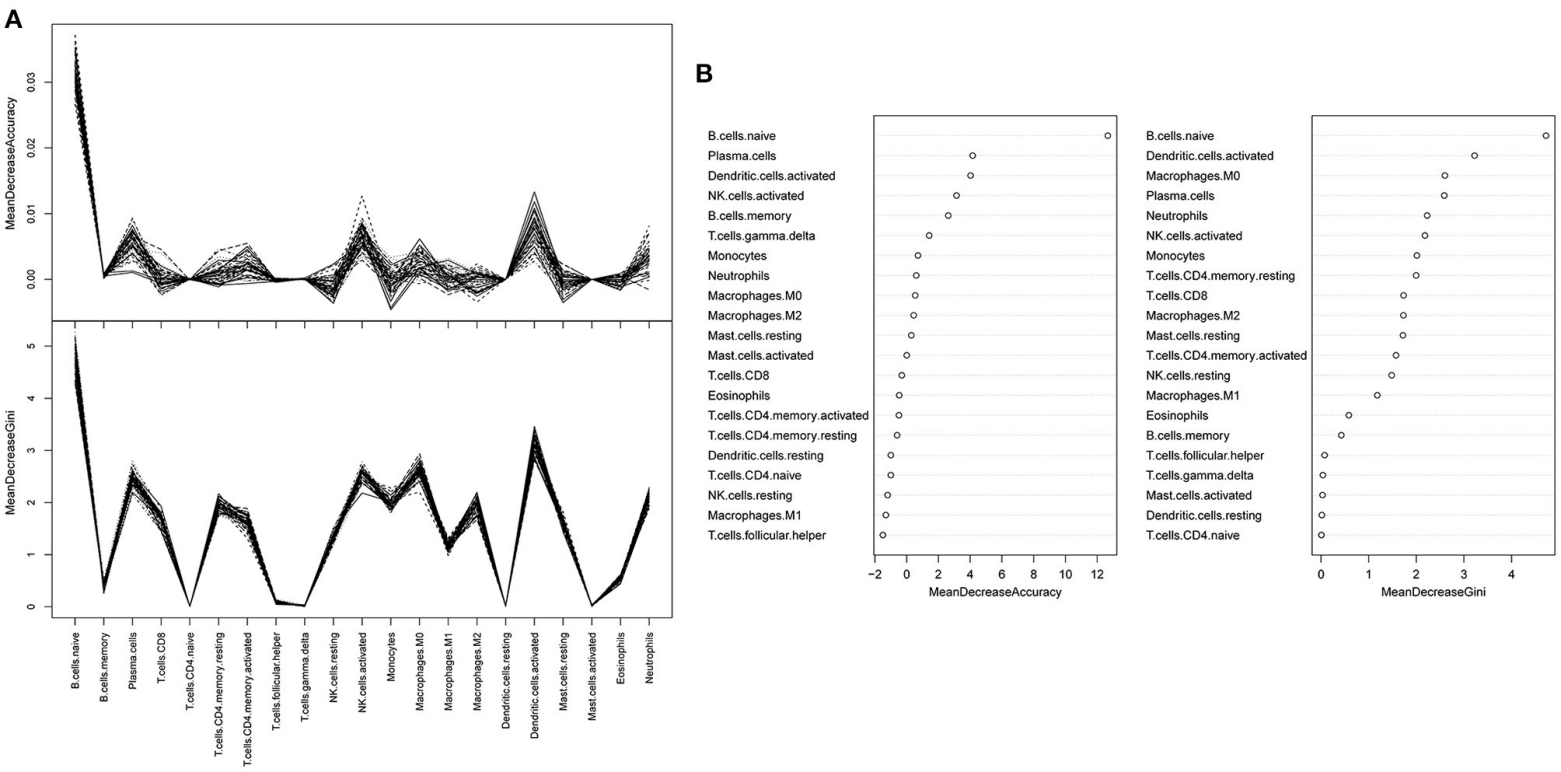
A random forest analysis was trained to estimate the important values of immune infiltration class. **(A)** Random forest classifier analysis. The y-axis represented the mean decreased accuracy and mean decreased gin score, while the x-axis represented immune cell types. **(B)** The mean decreased accuracy of immune infiltration subsets.

**Figure 4:**
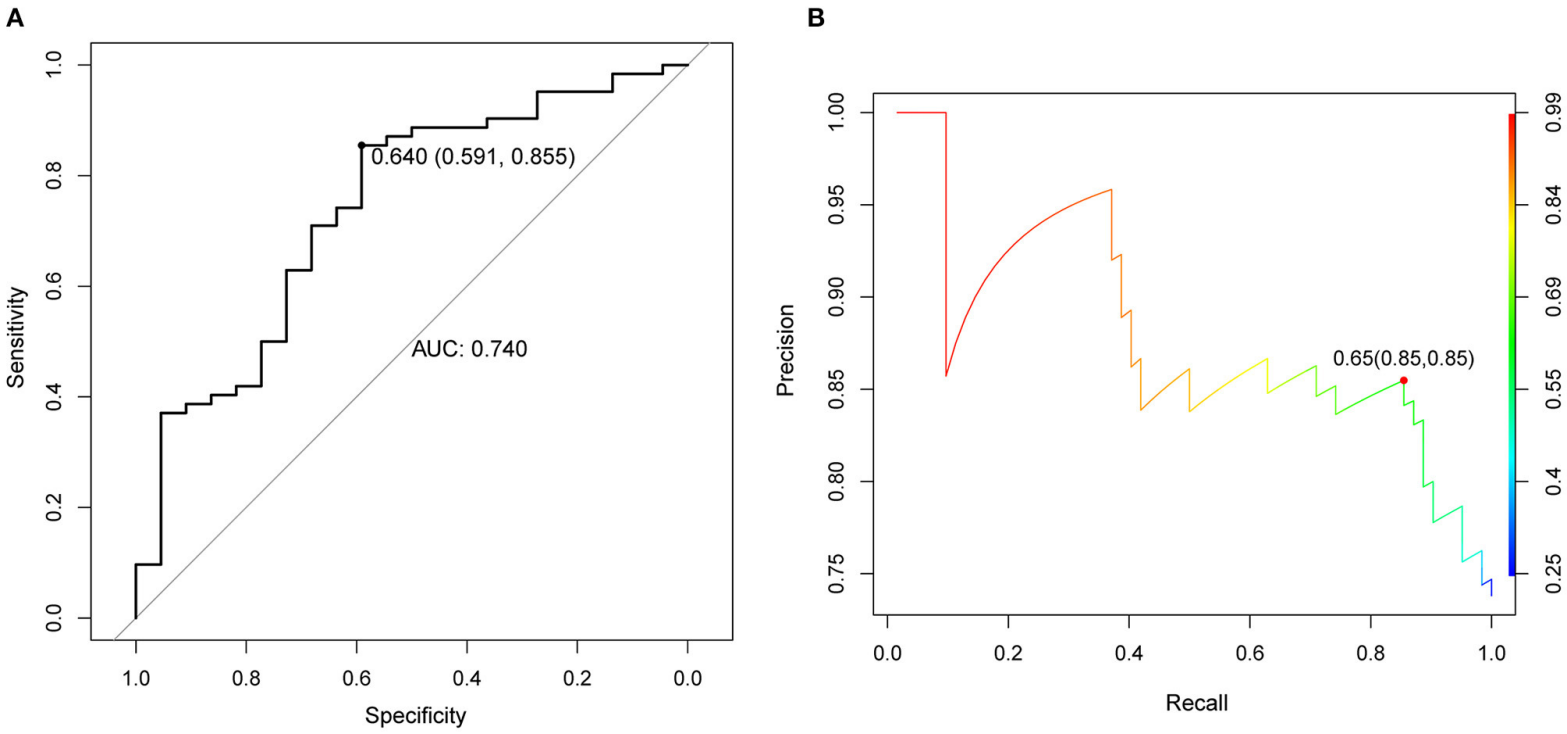
OOB cross validation results were visualized using receiver operating characteristic curve. **(A)** ROC curve, the horizontal axis represented the specificity of false positive rate (FPR), and the vertical axis represented the sensitivity of true positive rate (TPR). AUC as a numerical value could directly evaluate the quality of the model, the larger the value in the range of 0 to 1, the better. **(B)** The diagram of OOB error. OOB: out of bag.

A random forest classifier was trained on the microarray dataset using variable importance measures of 21 immune cells. Patients with intermediate or severe PH and non-PH were considered as the training set, while the 32 PH patients were considered as the testing set. We firstly ran random forest analysis 50 times to evaluate variable importance, including mean decreased accuracy and mean decreased gini. The results were visualized in Figures 3A,B, and the four immune cells with significantly different immune infiltration obtained a high score. A total of 13 immune cells with 500 trees were screened as major cluster to construct random forest classifier model. OOB cross-validation was performed, and the results are shown in Figure 4. The area under the receiver operating characteristic curve (AUC) value was 0.74 and the corresponding threshold for prediction was 0.64. As shown in the precision-recall diagram, the optimal cut point was 0.65. We ran the OOB cross-validation on the training set and obtained an accuracy of 79%. Finally, the validation was performed on the testing set and 22 specimens were identified as PH-positive samples (the accuracy was 69%), which indicated that this model was an effective approach for PH prediction. However, this model should be further substantiated in a larger cohort. Our findings further indicated that infiltrating immune cells in PF patients were associated with the PH status of patients.

## Discussion

In this study, we have explored the correlation between infiltrated immune cells in PF patients and differential PH status. Based on genomic expression profile analysis and CIBERSORT algorithm application, we found that accumulation of naive B cells and plasma cells was identified in PH patients compared with control non-PH samples. In contrast, activated DCs and M2 macrophages were relatively lower in PH patients. There was still a relatively high accuracy of random forest model after OOB cross-validation, which further evidenced that infiltrating immune cells in PF patients were associated with the PH status of patients.

Various types of infiltrated immune cells have been described in the pathogenesis of PF diseases, whereas the potential roles were not well-understood. Naive B cells are a type of B cells that have not been exposed to an antigen. They are originally generated in bone marrow and triggered by antigen binding. After being exposed to an antigen, the naive B cells can develop to become memory B cells or plasma cells that secrete antibodies (19). The reports of naive B cells in PH-associated PF patients were limited. So far, plasma B cells have been confirmed to be involved in the pathogenesis of PF. Mohammed et al. showed that B cells were critical for lung fibrosis control, and it promoted macrophage overexpression of prostaglandin-E2 in IL-9 transgenic mice (20). Recently, examinations of diseased lungs from PF patients revealed that highly B-cell aggregation was prevalent in pulmonary tissues (21, 22). Although B-cell infiltration varies among PF patients, it is possible that B-cell target therapies may be more consistent while patients exhibited a high CD20^+^ cell infiltration (23).

DCs are potent antigen-presenting cells. Accumulation of DCs has been found in lymphoid follicles of PF lung tissues. However, their pathogenetic relevance in PF patients with intermediate or severe PH status was poorly defined. Lung DCs acted as key pro-inflammatory cells to sustain pulmonary inflammation and fibrosis status in mice model (24). Resident cells in PF, such as fibroblasts and epithelial cells, can sustain chronic inflammation by driving the accumulation of DCs in lymphoid follicles (25). Recently, the data derived from Bantsimba-Malanda et al. (24) showed that increased accumulation of DC subsets was identified in lung tissue of PF mice model, and selective depletion of lung DCs can aggravate PF status, which indicated a regulatory role of lung DCs in PF disease. As for the role of DCs in PH status, perivascular inflammation existed in many types of PH, and inflammatory pathways were involved in multiple mediators, such as T cells, DCs, macrophages, and mast cells. Thus, immature DC accumulation was also described in remodeled pulmonary vessels, and it was involved in immunopathology in PH patients (7). Additionally, in our study, a random associated condition in a small part of PF patients was also a possible factor affecting the inflammation in PH. To our knowledge, our study firstly demonstrated a lower proportion of active DCs in PH associated with PF patients compared with non-PH patients. Together with previous studies, we suggested that abnormal infiltration of DCs acted as a key regulator in PF development, whereas as the disease progressed to a more severe PH status, the proportion of infiltrated DCs might be relatively decreased.

In addition to DCs, researchers have recognized the regulatory role of macrophage in PF pathogenesis over the past decades. Macrophages are small populations of leukocytes, and it can be activated to either classical M1 or alternative M2 phenotypes (26). In PF status, M1 macrophages express higher pro-inflammatory cytokines, while M2 macrophages express higher Th2-type cytokine levels. Since the PF was a pathological process of lung injury, a variety of immune cells interacted with regulatory cytokines affecting polarization of lung M1 and M2 macrophages (27). However, an increased number of perivascular macrophages were also found in vascular lesions of PH patients, and the recruitment of macrophage was associated with vascular remodeling in PH (28). Here, infiltration immune cell analysis results showed that M2 macrophages were relatively lower in PF patients with a more severe PH status, and these findings raised the evidence that abnormal proportion of macrophages might be a major factor in PF progression.

Additionally, inspired by previous research, we applied CICERSORT software in the exploration of correlation between immune cell infiltration and PH status, through which relative content of 22 immune cells were calculated concisely and quickly. At the same time, several limitations inevitably existed in our present research. Firstly, *via* the bioinformatic tools, absolute contents of immune cells could not be obtained, which still needs to be further studied in wet labs. The validation in single cells is lacking, adding which might improve reliability of our study. Besides, the present research cohort is limited, which might lead to some potential bias. In any case, the correlation between immune cell infiltration and PH status not only deserves further research but also provides a potential prediction tool.

## Conclusions

In conclusion, we have firstly presented the evidence that immune cell infiltration in PF patients was probably correlated with the PH status of patients. Abnormal accumulation of four types of immune cells were involved in development of PH, such as high proportions of naive B cells, plasma cells, and relatively low proportions of activated DCs and M2 macrophages. Our results might promote a potential method for PH prediction based on immune infiltration features.

## Data Availability Statement

Our data and related clinical information were retrieved from GEO database (number: GSE20129).

## Author Contributions

LF and XQ designed this research. FZ and LZ collected data. RH and JW analyzed data. ZY and LF wrote manuscripts. All authors read and approved the final manuscript.

## Conflict of Interest

The authors declare that the research was conducted in the absence of any commercial or financial relationships that could be construed as a potential conflict of interest.
