# Supplementary material for: Effect of Immune Cell Infiltration on Occurrence of Pulmonary Hypertension in Pulmonary Fibrosis Patients Based on Gene Expression Profiles
Source: Front Med (Lausanne). 2021 Jul 7;8:671617. doi: 10.3389/fmed.2021.671617 (PMC8292720; doi:10.3389/fmed.2021.671617)
Supplement: Supplementary file 1 [file Table_1.DOCX]

**Table S1. Demographic, Functional, and Perioperative Characteristics of Patients Included in the Study.**

| **Variable** | **Development Set (n=84)** | **Validation Set (n=32)** |
| --- | --- | --- |
| Age, y | 59±8 | 54±13 |
| Sex, male/female (% male) | 52/32 (62) | 21/11 (66) |
| BMI, kg/m | 26±4 | 26±4 |
| Smokers/never smokers, No. | 42/42 | 14/18 |
| Pack-y | 27±15 | 20±15 |
| UIP/non-UIP (% UIP) | 62/22 (74) | 21/11 (66) |
| Associated conditions, No. |  |  |
| Scleroderma | 6 | 4 |
| Sarcoidosis | 2 | 1 |
| Asbestosis | 2 | 0 |
| Rheumatoid arthritis | 2 | 0 |
| Silicosis | 0 | 1 |
| FVC, % pred | 54 ± 18 | 55 ± 17 |
| D lco , % pred | 41 ± 15 | 43 ± 16 |
| TLC, % pred | 61 ± 14 | 65 ± 18 |
| 6-min walking distance, m | 295 ± 94 | 289 ± 135 |
| mPAP, mm Hg | 29 ± 12 | 31 ± 17 |
| Pulmonary capillary wedge pressure,a mm Hg | 7 ± 3 | 6± 4 |
| Pulmonary hypertension ( ≥25 mm Hg), Yes/No (% Yes) | 52/32 (62) | 18/14 (56) |
| Pulmonary vascular resistance, dyn/s/cm | 530±387 | 497 ±437 |
| Cardiac output, L/min | 4.3 ±1.2 | 5.0 ± 1.2 |
| Severe pulmonary hypertension (≥40 mm Hg), Yes/No (% Yes) | 17/67 (20) | 7/25 (22) |
| Treatment, No. (% of total) |  |  |
| Prednisone alone | 28 (33) | 10 (31) |
| Prednisone+azathioprine | 18 (21) | 8 (25) |
| Prednisone+azathioprine+NAC | 14 (17) | 6 (19) |
| Prednisone+cyclophosphamide | 13 (16) | 5 (16) |
| No specifific treatment | 11 (13) | 3 (9) |

The pulmonary hypertension patients were diagnosed by right heart catheterization (RHC).
